# Cell-free fat extract promotes axon regeneration and retinal ganglion cells survival in traumatic optic neuropathy

**DOI:** 10.3389/fncel.2024.1344853

**Published:** 2024-03-07

**Authors:** Yiyu Sun, Di Chen, Tao Dai, Ziyou Yu, Hui Xie, Xiangsheng Wang, Wenjie Zhang

**Affiliations:** ^1^Department of Wound Reconstructive Surgery, Tongji Hospital Affiliated to Tongji University, School of Medicine, Tongji University, Shanghai, China; ^2^Department of Neurosurgery, The First Affiliated Hospital of Zhengzhou University, Zhengzhou, Henan, China; ^3^Shanghai Key Laboratory of Tissue Engineering, Department of Plastic and Reconstructive Surgery, Shanghai 9th People’s Hospital, Shanghai Jiao Tong University, Shanghai, China; ^4^Department of Geriatrics, Tongji Hospital, Tongji Medical College, Huazhong University of Science and Technology, Wuhan, Hubei, China; ^5^Department of Plastic and Reconstructive Surgery, Affiliated Hangzhou First People’s Hospital, School of Medicine, Westlake University, Hangzhou, Zhejiang, China

**Keywords:** cell-free fat extract, axon regeneration, RGCs survival, inflammation factors, microglia

## Abstract

Injuries to axons within the central nervous system (CNS) pose a substantial clinical challenge due to their limited regenerative capacity. This study investigates the therapeutic potential of Cell-free fat extract (CEFFE) in CNS injury. CEFFE was injected intravitreally after the optic nerve was crushed. Two weeks post-injury, quantification of regenerated axons and survival rates of retinal ganglion cells (RGCs) were performed. Subsequently, comprehensive gene ontology (GO) an-notation elucidated the cellular origins and functional attributes of CEFFE components. Molecular mechanisms underlying CEFFE’s therapeutic effects were explored through Western blotting (WB). Additionally, levels of inflammatory factors within CEFFE were determined using enzyme-linked immunosorbent assay (ELISA), and histological staining of microglia was conducted to assess its impact on neuroinflammation. CEFFE demonstrated a significant capacity to promote axon re-generation and enhance RGCs survival. GO annotation revealed the involvement of 146 proteins within CEFFE in axonogenesis and neurogenesis. WB analysis unveiled the multifaceted pathways through which CEFFE exerts its therapeutic effects. Elevated levels of inflammatory factors were detected through ELISA, and CEFFE exhibited a modulatory effect on microglial activation in the retinal tissue following optic nerve crush (ONC). The present study highlights the therapeutic promise of CEFFE in the management of CNS injuries, exemplified by its ability to foster axon regeneration and improve RGCs survival.

## 1 Introduction

The axon serves as a foundational structural and functional unit of a neuron, pivotal in the transmission of signals and substances throughout the body. After CNS injury, lesioned axons fail to regenerate, which leads to compromised functional recovery (Fischer and Leibinger, 2012; Blennow et al., 2016). These challenges can manifest as conditions such as blindness or paralysis. The optic nerve crush (ONC) model is one of most predominant method to simulate axon injury in CNS—given that the retina is categorically a part of the CNS (Gennarelli et al., 1989; Williams et al., 2020). More importantly, the mechanism of injury has been verified by the optic nerve crush model and translated to other axon injury models, including those of spinal cord injury.

In recent years stem cells have been shown to promote axon regeneration (Mesentier-Louro et al., 2014; Park et al., 2021). Among various stem cell classifications, adipose-derived stem cells (ADSCs) are favored because of their accessibility, reduced immunogenicity, and multiple differentiation potential. Stem cells may facilitate therapeutic applications by both supplanting native cells and discharging trophic factors. Yet, existing literature lacks robust evidence to suggest that ADSCs can differentiate directly into RGCs. ADSCs contain many trophic factors, such as the vascular endothelial growth factor (VEGF), brain-derived neurotrophic factor (BDNF) and hepatocyte growth factor (HGF). Exogenously applied trophic factors, such as Ciliary neurotrophic factor (CNTF) or HGF, have been observed to stimulate regeneration in injured axons. Concurrently, certain studies suggest that the synergistic application of multiple trophic factors yields superior outcomes compared to monotherapeutic approaches (Logan et al., 2006).

In prior investigations, we pioneered a novel methodology to generate cell-free fat extract (CEFFE). CEFFE is an acellular fluid extracted from human subcutaneous adipose tissue without cellular components and lipid residues and has shown no immunogenicity, good biosafety. Our previous research has demonstrated the robust potential of CEFFE in tissue repair and regeneration, such as skeletal, skin and reproductive systems (Deng et al., 2020; Jia et al., 2022; Liu et al., 2022). However, the effect of CEFFE on CNS injury remains unknown. Since the composition of CEFFE is similar to stem cell paracrine, we hypothesized that CEFFE might possess the potential to promote axon regeneration akin to stem cells. To test this hypothesis, this study evaluated the effects of CEFFE on axon regeneration in ONC model and investigated the underlying mechanisms.

## 2 Materials and methods

### 2.1 CEFFE preparation

The preparation of the CEFFE adhered to established protocols (Yu et al., 2018; Deng et al., 2020; Xu et al., 2020). Briefly, human adipose tissue was acquired from healthy liposuction donors. The tissue was rinsed with saline to remove red blood cells and centrifuged at 1200 × *g* for 3 min. The upper oily and lower fluid layers were then discarded, and the middle fat layer was harvested and mechanically emulsified. The emulsified fat was then frozen at −80°C for 24 h and thawed at 37°C for 30 min to further disrupt the cells in the fat tissue. After one cycle of the freeze/thaw process, the fat was centrifuged again at 2000 × *g* for 5 min and separated into four layers, and the third aqueous layer containing the CEFFE was collected and frozen at −80°C for experimental purposes. The final extract was passed through a 0.22-μm filter (Corning Glass Works, Corning, NY, USA) for sterilization and removal of cell debris. The CEFFE protein concentrations were measured, using a Pierce BCA Protein Assay Kit (Thermo Fisher Scientific, Waltham, MA, USA). The study was approved by the Ethics Committee of the Shanghai Jiaotong University School of Medicine, Shanghai, China (SH9H-2018-T22-1).

### 2.2 Surgical procedures

C57 BL/6 mice aged 5 weeks were used in this experiment. All surgical procedures were conducted under anesthesia, with 1% pentobarbital sodium (10 μl/g) administered intraperitoneally. After surgery, ointment containing ophthalmic antibiotic was applied to protect the cornea. Mice were kept on a 12-h light/dark cycle with free access to food and water. Mice with opaque lenses were excluded from the experiment. The study was approved by the Ethics Committee of the Shanghai Jiaotong University School of Medicine, Shanghai, China.

Subsequent to anesthesia administration, the left optic nerve was exposed intraorbitally. The optic nerve was crushed with forceps (Fine Science Tools, 11480-11) for 5 s, 1 mm distal to the eyeball. Care was taken to avoid damaging the underlying ophthalmic arteries. Immediately after crushing, a fine glass micropipette was inserted into the posterior chamber of the left eye and avoid damaging the lens. Before injection, approximately 3 μl vitreous humor was pumped out. Subsequently, using a Nanoliter 2010 injector (WPI, NL2010MC2T), 3 μl of the CEFFE (5 μg/μl), Phosphate buffered saline (PBS) or CNTF (5 μg/μl, R&D system, 257-NT-050) were slowly injected into the vitreous chamber at the appropriate time points, in accordance with the needs of the experiments. The needle was removed after 5 min until the solution completely diffused.

To label regenerating axons, 2 μl of Alexa-555 conjugated cholera toxin β subunit (CTB-555) (2 μg/μl, C22843, Thermo Fisher) was injected into the vitreous with a Hamilton syringe (80,030, Hamilton) 2 weeks later after the initial injury.

### 2.3 Immunohistochemistry

Mice were humanely sacrificed and intracardially perfused with a cold saline solution, followed by a PBS solution containing 4% Paraformaldehyde (PFA). The eyes and optic nerves were carefully removed from the connective tissue, postfixed for 2 h in 4% PFA solution at 4°C, and subsequently transferred to 30% sucrose for a 48-h incubation at 4°C. For section staining, the retinal sections were incubated with primary antibodies CD11b (1:200, Abcam ab226482) overnight at 4°C and washed three times for 15 min each with PBS. Secondary antibodies were then applied and incubated for 1 h at room temperature. For whole-mount retina staining, the retina was cut into petals and blocked in PBS containing 5% normal goat serum and 0.4% Triton X-100 for 2 h at room temperature. Thereafter, the tissues were incubated with primary antibody Neuronal Class III β-Tubulin (Tuj1) (1:500, Abcam ab18207) in a blocking buffer overnight at 4°C. After three rinses with PBST (1x Phosphate-Buffered Saline, 0.1% Tween 20), retinas were incubated with the secondary antibody goat anti-rabbit Alex-488 (1:1000 dilution, Thermo Fisher R37116). Following three washes with PBST, the tissues were sealed for observation. The optic nerve was embedded in O.C.T. Compound (25608–930, SAKURA) and longitudinal sections (14 μm) were prepared and stored at −20°C until further use.

### 2.4 Quantification of axons, RGCs and microglia

The number of CTB-labeled axons was counted at different distances from the crush site (250, 500, 750, 1,000, and 1,500 μm) in four sections per nerve. Each group included six mice. The cross-sectional width of the nerve was measured at the point at which the counts were taken and used to calculate the number of axons per millimeter of nerve width. The number of axons per millimeter was then averaged over all sections. In addition, Σa_d_, the total number of axons extending distance d in a nerve with radius r, was estimated by summing over all sections with thickness t (14 μm), as follows (Park et al., 2008): Σa_d_ = πr^2^ x [average axons/mm]/t.

For RGCs counting, whole mount retinas were immunostained with the Tuj1 antibody and six to nine fields were randomly sampled from each retina. The percentage of RGCs survival was calculated as the ratio of the number of surviving RGCs in the crushed eyes to that in the contralateral uninjured eyes. Counts of surviving cells and regenerated axons were performed by a single observer who was blinded to the surgical manipulations and treatments.

For microglia quantification, the magnitude of CD11b + microglia activation was defined as the number of CD11b + cells present in the total area. Four to five slices of retina per mouse were randomly selected to count the total number of CD11b + cells and the number of CD11b + cells present in the GCL (ganglion cell layer) layer.

### 2.5 Proteomics analysis

The CEFFE samples were processed for quantitative proteomic analysis as previously described (Yu et al., 2018). Briefly, protein concentration of CEFFE was measured, using a BCA protein assay kit. The samples were then digested with trypsin for subsequent Liquid chromatography–tandem mass spectrometry analysis (LC-MC). Gene ontology (GO) analysis was performed to classify all identified protein types into three categories (cell component, molecular function, and biological process), using the UniProt-GOA database,^1^ InterProScan,^2^ and GO annotation.^3^ Proteins related to axon genesis and neurogenesis were also identified.

### 2.6 Western blot assays

Three mice per condition were sacrificed by cervical dislocation and retinas were dissected, homogenized, and solubilized at 4°C in a cell lysis buffer (P0013, Beyotime) for 30 min, supplemented with 1 mM phenylmethylsulfonyl fluoride, 50 mM sodium fluoride, 1 mM Na3VO4, and a protease inhibitor. Total protein lysates were separated by sodium dodecyl sulfate polyacrylamide gel electrophoresis and analyzed by western blotting with anti-mTOR (Merck SAB2702297, dilution 1:1000), anti-ROCK2 (Santa Cruz sc-398519, dilution 1:1000), anti-cleaved calpain (Abcam ab92333, 1:1000) and anti-β-actin (Beyotime AF5001, 1:1000). Data analysis was performed using the NIH ImageJ software. The mean density of each band was normalized to that of the β-actin signal in the same sample.

### 2.7 Enzyme-linked immunosorbent assay

Enzyme-linked immunosorbent assays (R&D Systems company, USA) were performed, according to standard protocols, to quantify the levels of transforming growth factor beta 1 (TGFβ-1), tumor necrosis factor alpha (TNFα), interleukin 4 (IL-4), and interleukin 1 beta (IL-1β) in CEFFE. The antibodies used were as followed: Human/Mouse/Rat/Porcine/Canine TGF-beta 1 Quantikine ELISA, Catalog #: DB100C; Human IL-4 Quantikine HS ELISA Kit, Catalog #: HS400; Human IL-1 beta/IL-1F2 Quantikine HS ELISA Kit, Catalog #: HSLB00D; Human TNF-alpha Quantikine ELISA Kit, Catalog #: DTA00D.

### 2.8 Quantitative real-time PCR

To determine the levels of expression of the pro-inflammation factors (IL-1β, IL-6, inducible nitric oxide synthase (iNOS), and TNF-α), anti-inflammation factors (IL-10, arginase-1 (ARG), TGF-β, and CD-206), treated retina mRNA was extracted using the Total RNA Extraction Reagent (EZBioscience, Roseville, USA) according to the manufacturer’s instructions. Briefly, 1 μg total RNA was reverse transcribed to cDNA using a reverse transcription master mix (EZBioscience, Roseville, USA) according to the manufacturer’s instructions. Subsequently, qRT-PCR was conducted using an SYBR Green qPCR master mix (ROX2 plus; EZBioscience, Roseville, USA). Cycling parameters were 95°C for 5 min, then 40 cycles at 95°C for 10 s followed by 60°C for 30 s. At least three technical replicates were performed for each sample. Relative expression levels were calculated using the 2^−ΔΔCq^ method and are presented as fold-change relative to the glyceraldehyde 3-phosphate dehydrogenase house gene expression. Primers for RT-qPCR are listed in Supplementary File 2: Table S1.

### 2.9 Statistical analysis

Statistical analysis and graph creation were performed with GraphPad Prism 6 (GraphPad Software). Data were presented as the mean ± standard error of mean. We used analysis of variance with Tukey’s test to adjust for multiple comparisons among more than two groups. Differences were considered statistically significant at *p*-values of <0.05.

## 3 Results

### 3.1 Characterization of CEFFE

The CEFFE was obtained from the lipoaspirate of six healthy female volunteers (The mean age was 31 years, range 24–36 years) and was produced, as previously described (Yu et al., 2018). Briefly, approximately 7 ml of pinkish CEFFE was obtained from 50 ml of centrifuged lipoaspirate (collected after the first spin). The total CEFFE protein concentration was 4745.43 ± 751.73 μg/ml (*n* = 6).

### 3.2 CEFFE promotes axon regeneration

**Figure 1:**
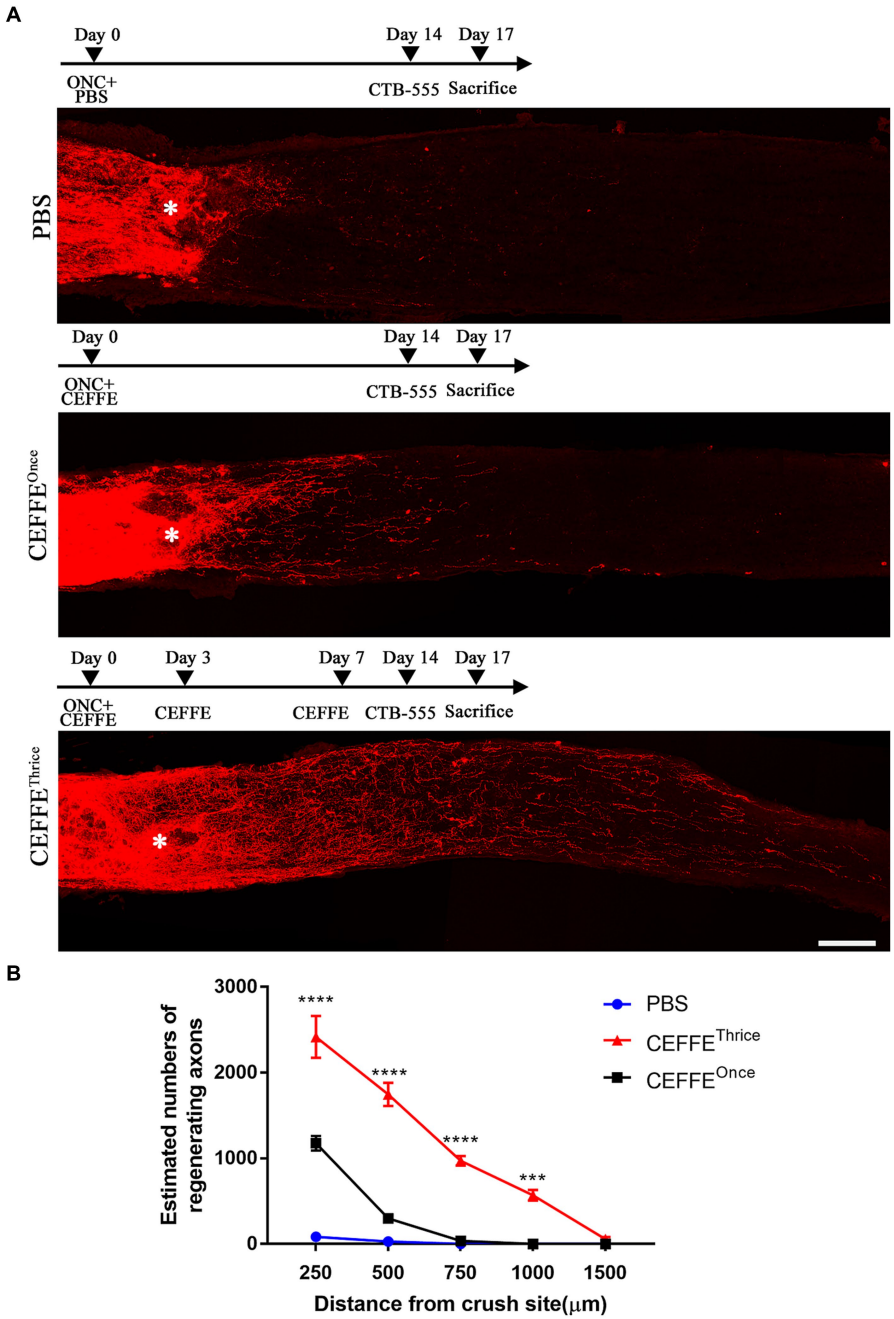
CEFFE promotes axon regeneration. **(A)** Confocal images of optic nerve longitudinal sections showing regenerating fibers labelled with CTB-555. * Represents crush site. Scale bar, 100 μm. **(B)** Quantification of regenerating axons at different distances distal to the lesion site. ***p < 0.001, ****p < 0.0001. Data are presented as means ± standard error of mean, *n* = 6 mice per group. Two-way analysis of variance with Tukey’s test.

A single injection of the CEFFE exhibited limited effect on axon regeneration, only a few fibers extended beyond the crush site. The number of regenerated axons was 1,176 at 250 μm from the crush site and sharply decreased to 298 at 500 μm. At 750 and 1,000 μm the number was 37 and 0, respectively. Since injured RGCs underwent several pathological phases until death (Garcia-Valenzuela et al., 1994). Within 6 h after injury, the number of RGCs did not decrease. On days 3–7 post-injury, the rate of RGCs increased gradually, peaking on day 7. So, we adapted our protocol and injected the CEFFE at day 0, day 3 and day 7 after ONC. As a result, robust axon regeneration was observed (Figure 1A). The number of regenerated axons at different distances in the CEFFE-thrice-injected group was significantly higher than that in the CEFFE-once-injected group, with counts of 2,417, 1745, 970, 569, and 56, respectively. The number of regenerated axons gradually declined, commencing from the lesion site, and the longest regenerated axons reached approximately 1.5 mm from the crush site. These results underscore the capacity of repeat CEFFE administration to promote axon regeneration.

### 3.3 CEFFE is better than a single neurotrophic factor for axon regeneration

**Figure 2:**
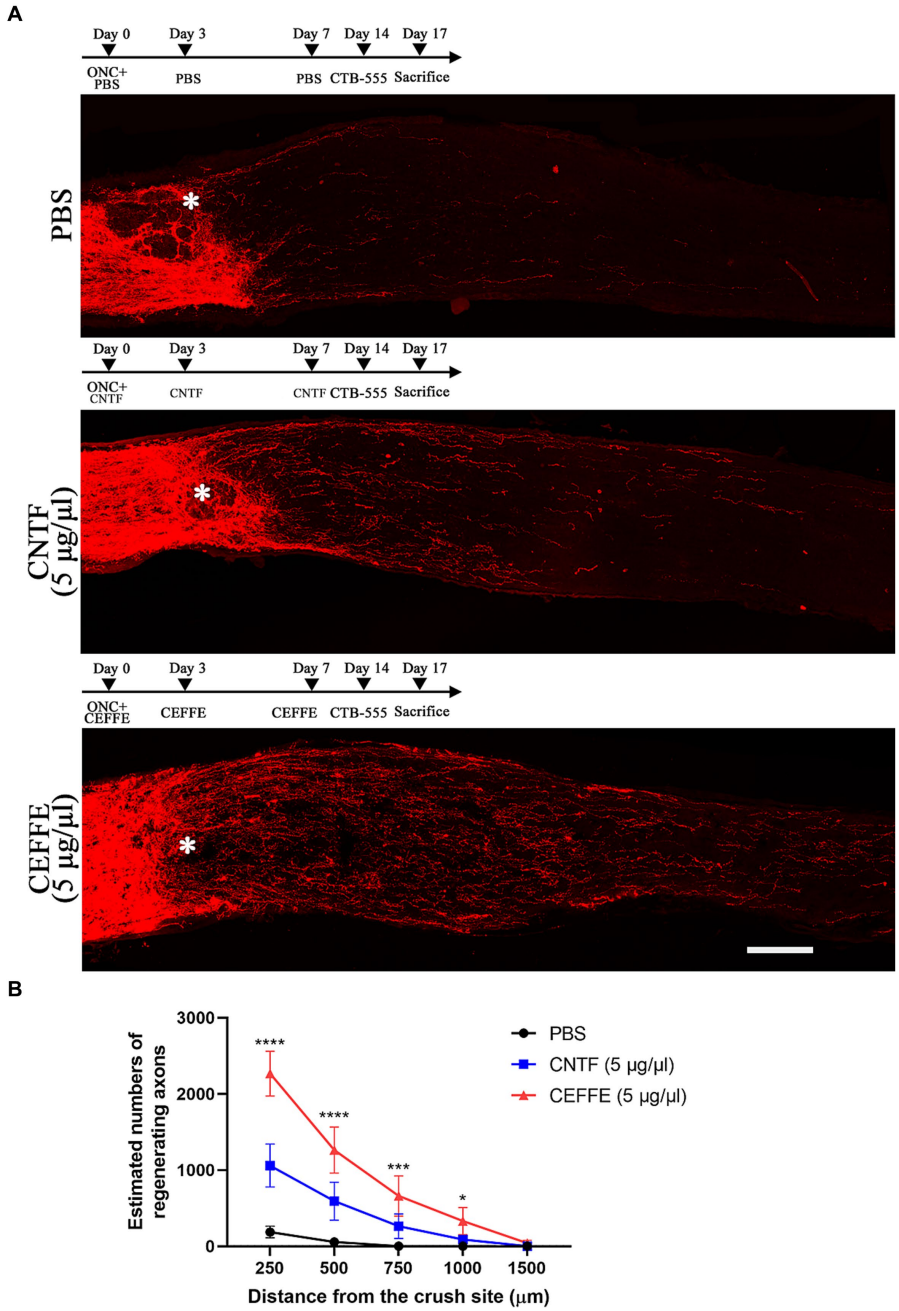
CEFFE induces more robust axon regeneration than CNTF. **(A)** Confocal images of optic nerve sections showing regenerating axons labeled by CTB-555 2 weeks after optic nerve injury from PBS, CNTF- (5 μg/μl), and CEFFE-treated (5 μg/μl) eyes. * Represents crush site. Scale bar, 100 μm. **(B)** Quantification of regenerating axons at different distances distal to the lesion site. Data are presented as mean ± standard error of mean, *n* = 6 mice per group. *p<0.05, ***p<0.001, ****p < 0.0001, Two-way analysis of variance with Tukey’s test.

Both CEFFE and CNTF were intravitreally injected on days 0, 3, and 7 post-crush (Figure 2). When utilizing CNTF (5 μg/μl), it was observed to induce axon regeneration to a certain extent. The counts of regenerated axons in the CNTF-treated group were 1,060, 593, 263, 88, and 2 at different distances from the crush site, respectively. However, under identical concentration conditions (5 μg/μl), the number of regenerated axons in the CEFFE-treated group significantly exceeded that of the CNTF-treated group. Specifically, the counts were 2,268, 1,264, 660, 331, and 41 in the CEFFE-treated group. These results, as depicted in Figure 2, clearly demonstrate that the impact of the CEFFE on axon regeneration surpassed that of a single trophic factor.

### 3.4 CEFFE promotes RGCs survival

**Figure 3:**
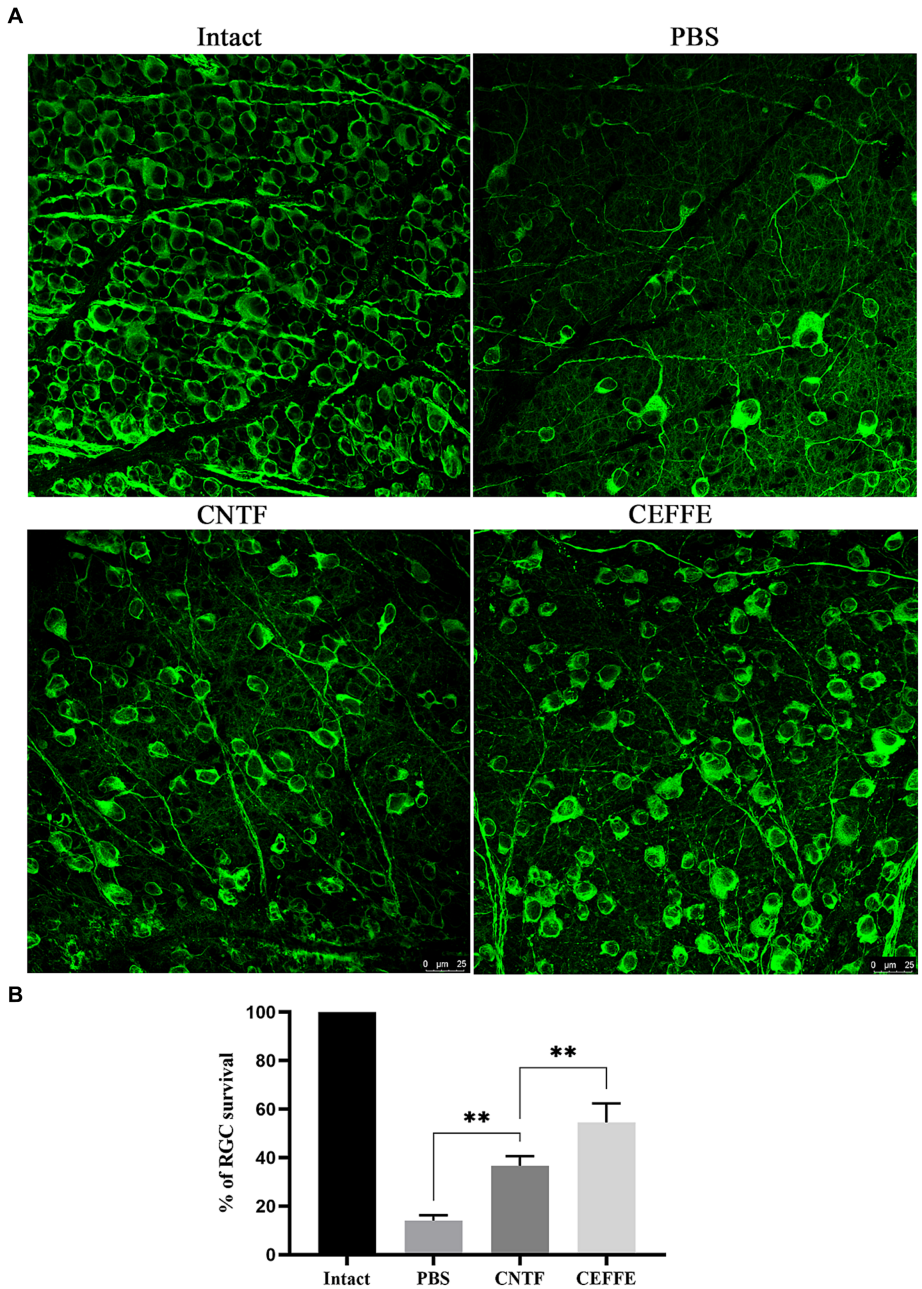
CEFFE promotes RGCs survival after optic nerve crush. **(A)** Confocal images of retinal whole-mounts show surviving Tuj1+ RGCs at 2 weeks after optic nerve injury. Scale bar, 25 mm. **(B)** Quantification of RGCs survival at 2 weeks after injury, expressed as a percentage of total Tuj1+ RGCs in the uninjured retina. Data are presented as the mean ± standard error of mean, *n* = 3 mice per group. **p < 0.01, One-way analysis of variance with Tukey’s test.

In addition to promoting axon regeneration, we found that the CEFFE had a significant protective effect on RGCs survival. In contrast to 14.37% of RGCs surviving rate in the PBS-treated group, approximately 54.5% of RGCs survived after the CEFFE injection (see Figure 3), which was significantly higher than CNTF-treated group (36.61%). These results indicate the neuroprotective effects of CEFFE.

### 3.5 CEFFE comprises multiple factors related to axongenesis and neurogenesis

To gain a better understanding of CEFFE, we analyzed its components. We have previously reported detailed bioinformatics information (including total protein levels and GO classification) of CEFFE (Yu et al., 2018). In this study, delved deeper into the proteomic data, specifically identifying and categorizing proteins related to neurogenesis or axongenesis. We found 146 proteins involved in axongenesis or neurogenesis and sorted out according to subcellular localization (see Supplementary File 1: Table S1). Among which, Signal transducer and activator of transcription 3 (STAT3), Ras-related C3 botulinum toxin substrate 1 (RAC1), and cell division control protein 42 homolog (CDC42), have been shown to regulate axon regeneration (Lorenzetto et al., 2013; Namekata et al., 2014; Mehta et al., 2016). Since the regeneration of damaged axons share some similar processes as does axon growth during development (He and Jin, 2016; Mahar and Cavalli, 2018), we speculated that these factors in CEFFE may contribute to axon regeneration and provide neuroprotection.

### 3.6 CEFFE promotes axon regeneration and RGCs survival from diverse aspects

To further explain the mechanism underlying the regeneration phenomenon, we conducted experiments aimed at monitoring changes within retina. Firstly, we perform ELISA experiment to examine the expression of inflammation factors in CEFFE. Studies have demonstrated that elevated levels of pro-inflammatory cytokines such as IL-1β and TNFα are neurotoxic and closely associated with substantial axonal damage (Kitaoka et al., 2017; Mou et al., 2021). Conversely, the anti-inflammatory factors TGFβ-1 and IL-4 have been suggested to promote axonal growth during the inflammatory response (Andries et al., 2020). The mean level and variation of pro-inflammatory (TNFα and IL-1β) and anti-inflammatory factors (TGFβ-1and IL-4) were summarized in Table 1.

**Table 1:** Mean inflammation factors concentration in CEFFE (*n* = 6, pg./ml).

	TGFβ-1	IL-4	TNFα	IL-1β
Mean	492.01	15.70	48.9	0.9
SEM	195.22	6.55	13.49	1.37

**Figure 4:**
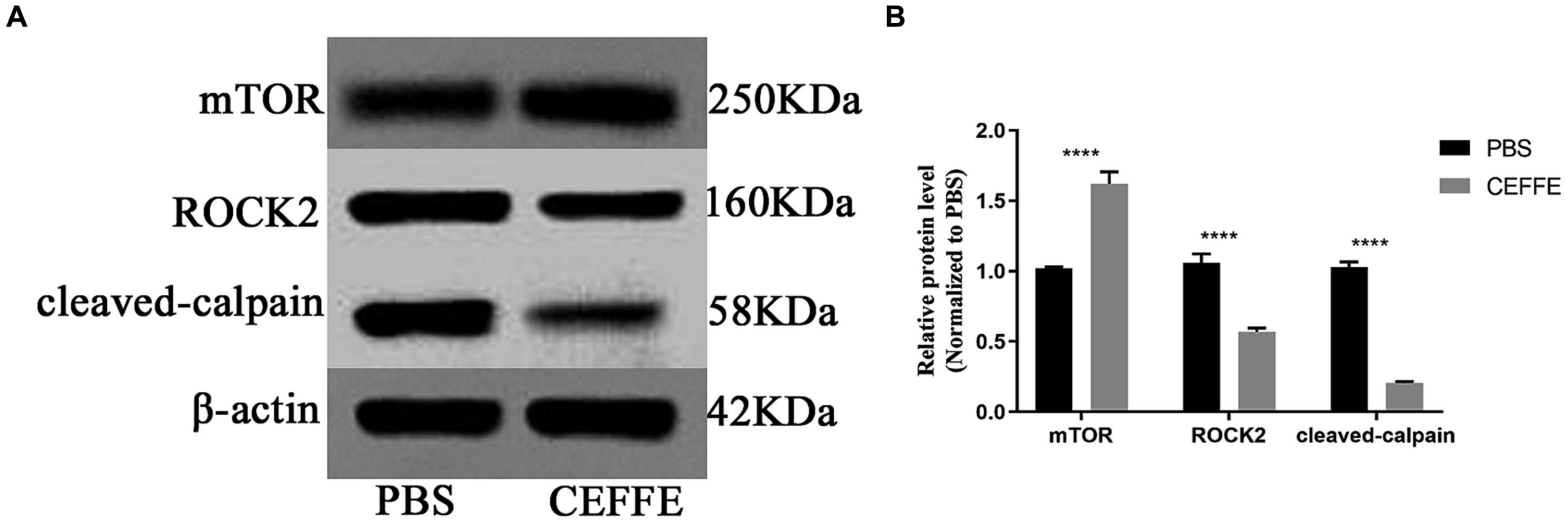
CEFFE modulates apoptosis and metabolism in retina. **(A)** Western blots detected the expression of mTOR, ROCK2 and cleaved-calpain in retinal lysates from PBS-treated and CEFFE-treated groups. **(B)** Quantification of band intensities normalized to β-actin as a loading control and relative to that of PBS are depicted (bars represent mean ± standard error of mean, *n* = 3 mice per group). ****p < 0.0001, Two-way analysis of variance with Tukey’s test.

Considering the well-documented role of mTOR in regulating anabolic metabolism and its potential significance in supporting axon regenerative growth in injured neurons (Park et al., 2008; Lu et al., 2014; Curcio and Bradke, 2018), we investigated the expression of mTOR. Conversely, we also examined the expression of ROCK2, a known inhibitor of axon regeneration (McKerracher et al., 2012). and cleaved-calpain as an indicator of apoptosis in the retina. As shown in Figure 4, compared with PBS treated group, the expression of mTOR was about 1.6-fold in CEFFE-treated group. While the expression of ROCK2, and cleaved-calpain reduced to 0.5 and 0.2-fold, respectively.

**Figure 5:**
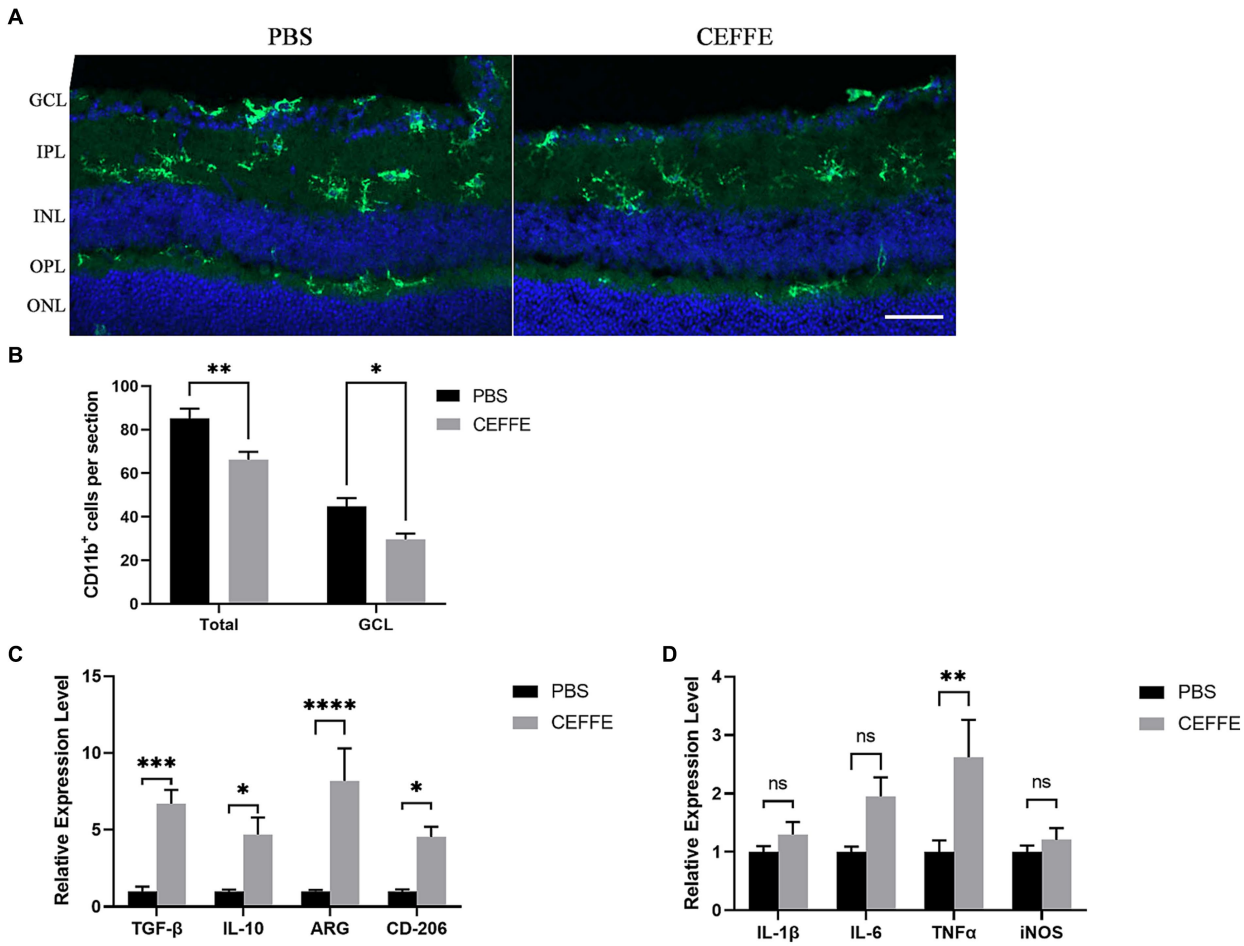
CEFFE attenuates microglia activation and modulate inflammation factors in retina. **(A)** Representative confocal pictures of sections showing CD11b-positive microglia (green) in retina. (GCL, ganglion cell layer; IPL, inner plexiform layer; INL, inner nuclear layer; OPL, outer plexiform layer; ONL, outer plexiform layer). Scale bar, 50 μm. **(B)** Quantification of CD11b-positive cells in GCL and entire retina. **(C)** Quantification of ARG, IL-10, CD206, and TGF-β mRNA expression by RT-qPCR. **(D)** Quantification of IL-1β, IL-6, iNOS, and TNF-α mRNA expression by RT-qPCR. One-way ANOVA and Tukey’s multiple comparisons test. *p < 0.05, **p < 0.01, ***p < 0.001, ****p < 0.0001 (*n* = 5 mice per group).

At last, our observations indicated that CEFFE exhibited an inhibitory effect on microglia activation (see Figures 5A,B). The use of CD11b to label activated microglia revealed that the total number of CD11b + cells was 85.2 ± 4.4 in the PBS-treated group, but notably reduced to 66.2 ± 3.59 in the CEFFE-treated group within the entire retina section. This decline in the number of activated microglia was significant. Additionally, in GCL, when compared to the control group (44.8 ± 3.76), the number of activated microglia in the CEFFE-treated group was significantly reduced to 29.6 ± 2.71. To further explore the potential role of CEFFE in resolving inflammation, we conducted RT-qPCR for proinflammatory and anti-inflammatory factors in retina (see Figures 5C,D). Our results showed that the mRNA expression of anti-inflammatory factors (ARG, IL-10, CD-206 and TGF-β) were remarkably elevated. In contrast, except for TNF-α, the changes of pro-inflammatory factors (IL-1β, IL-6 and iNOS) were not statistically significant. These findings collectively provide insights into the multifaceted mechanisms underlying CEFFE’s regenerative and neuroprotective effects.

## 4 Discussion

The secretome from the ADSCs has been used in pre-clinical studies of traumatic brain injury (Tajiri et al., 2014; Jha et al., 2018; Patel et al., 2018). Moreover, in some studies, the secretome from the ADSCs emerged as superior to that from the BMSCs in the treatment of neurodegenerative diseases (Katsuda et al., 2013; Lee et al., 2016). Researcher believed that its neuroprotective role is primarily mediated through paracrine mechanism, and found effective axon regeneration needs a synergistic interplay among various neurotrophic factors (Huang and Reichardt, 2001; Logan et al., 2006; Leibinger et al., 2016). CEFFE is a mixture of diverse proteins, including trophic factors, inflammation factors, and chemokines. The findings depicted in Figure 1 corroborate the efficacy of CEFFE in promoting axon regeneration. To ascertain the superior performance of CEFFE in comparison to a single trophic factor, we employed CNTF, one of the most typical trophic factors involved in axon regeneration (Müller et al., 2007), as a control. At the same condition, CEFFE showed more robust ability in axon regeneration compared with CNTF. It is plausible that a convergence of these factors acted in concert to activate signaling pathways vital to axon regeneration. For instance, both HGF and BDNF possess the capability to stimulate the mTOR pathway (Segarra et al., 2006; Lima Giacobbo et al., 2019). As the results shown in Figure 4, CEFFE increased mTOR expression and decreased ROCK2 expression.

Promoting neuronal survival and axon regeneration are two basic strategies to repair injuries in mature CNS. While modulation of intrinsic regulators can markedly alter the regenerative potential of neurons, singular gene manipulation can sometimes introduce incongruence in axon regeneration or neuronal survival. This has been exemplified in the cases of Armcx and Sox11 (Belin et al., 2014; Li et al., 2018). The evidence presented in Figure 3 demonstrates that CEFFE effectively safeguards RGCs from apoptosis. Moreover, the data in Figure 4 indicates a reduction in the expression of cleaved-calpain, a molecule associated with RGCs apoptosis (Cheng et al., 2018). In conjunction with the findings in Figure 1, our research posits that employing CEFFE can concurrently support both axon regeneration and the survival of RGCs.

Unlike the secretome of the ADSCs, CEFFE comprise both bioactive extracellular and intracellular components, as shown by our proteomics findings (see Supplementary File 1: Table S1). That may explain why the concentration of some trophic factors in CEFFE is much higher than that in the secretome from stem cells (Ribeiro et al., 2012; Johnson et al., 2014; Martins et al., 2017). For example, the concentration of the BDNF in CEFFE was approximately 1860.99 pg./ml, while that reported in other studies was approximately 13.22 or 37.03 pg./ml (Johnson et al., 2014; Martins et al., 2017; Yu et al., 2018). In addition to trophic factors, CEFFE exhibits considerably higher concentrations of inflammatory factors, as detailed in Table 1, in contrast to the mesenchymal stem cell secretome (Johnson et al., 2014). Intraocular inflammation promotes RGCs survival and axon regeneration (Leon et al., 2000; Benowitz and Popovich, 2011). However, persistent chronic inflammation will ultimately lead to RGCs death and axon degeneration. CEFFE demonstrates a relatively rapid *in vivo* degradation profile, and the anti-inflammatory factors (TGFβ-1 and IL-4) in CEFFE are more dominant in content than the pro- inflammatory factors (TNFα and IL-1β). This observation suggests that repeated, short-acting inflammatory stimulation elicited by CEFFE injections may explain the possible therapeutic mechanism of CEFFE from another perspective.

In another aspect, microglia, which serve as long-lived resident macrophages within the retinal parenchyma, undergo progressive activation subsequent to optic nerve injury. This activation process culminates in the production of pro-inflammatory cytokines and the generation of reactive oxygen species, both of which exhibit neurotoxic effects upon RGCs. Studies have proven that diminishing microglial activation, as observed in glaucoma and other CNS models, reduces neuronal cell death and mitigates neuroinflammation (Bosco et al., 2008; Takeda et al., 2018). Owing to its lack of immunogenicity and antioxidant effects, CEFFE is promising for relieve chronic inflammation. Our results showed that CEFFE could attenuate microglia activation after optic nerve crush (see Figure 5). The changes of anti and pro-inflammation factors indicated that after treated with CEFFE the microglia in retina presented an anti-inflammation state. The controlled microglia activation caused by CEFFE may contribute to axon regeneration and RGCs survival furtherly.

This study is not without its limitations. Although our results suggest that CEFFE operates as a cohesive unit, we must acknowledge the intricacies and vast network of factors involved. As a consequence, pinpointing the precise combination of factors within CEFFE that influence axon regeneration and the survival of RGCs has remained elusive. It is plausible that the effects of CEFFE encompass several intricate mechanisms, and isolating the principal mode of action proves challenging. To provide more comprehensive insights into the therapeutic potential of CEFFE, further investigations are warranted. Future studies should seek to delineate the temporal dynamics of CEFFE’s action *in vivo* and endeavor to optimize the selection of its active constituents. In summary, our study indicates that CEFFE can enhance axon regeneration and improve RGCs survival after optic nerve crush. As an allogeneic biological agent, CEFFE has the advantage of no cell component, no immunogenicity, etc. Therefore, it represents a potential therapeutic avenue for CNS injury.

## Data availability statement

The original contributions presented in the study are included in the article/supplementary material, further inquiries can be directed to the corresponding authors.

## Ethics statement

The studies involving humans were approved by Institutional Ethics Committee of Shanghai Ninth People’s Hospital, Shanghai Jiaotong University School of Medicine. The studies were conducted in accordance with the local legislation and institutional requirements. The participants provided their written informed consent to participate in this study. The animal study was approved by Institutional Ethics Committee of Shanghai Ninth People’s Hospital, Shanghai Jiaotong University School of Medicine. The study was conducted in accordance with the local legislation and institutional requirements.

## Author contributions

YS: Data curation, Formal analysis, Methodology, Writing – original draft. DC: Data curation, Formal analysis, Methodology, Writing – original draft. TD: Software, Writing – review & editing. ZY: Software, Writing – review & editing. HX: Writing – review & editing. XW: Conceptualization, Writing – original draft. WZ: Conceptualization, Funding acquisition, Writing – review & editing.

## Footnotes

^1^http://www.ebi.ac.uk/GOA/

^2^http://www.ebi.ac.uk/interpro/

^3^http://geneontology.org/
