# Supplementary figures and images for: Cell-free fat extract promotes axon regeneration and retinal ganglion cells survival in traumatic optic neuropathy
Source: Front Cell Neurosci. 2024 Mar 7;18:1344853. doi: 10.3389/fncel.2024.1344853 (PMC10954833; doi:10.3389/fncel.2024.1344853)

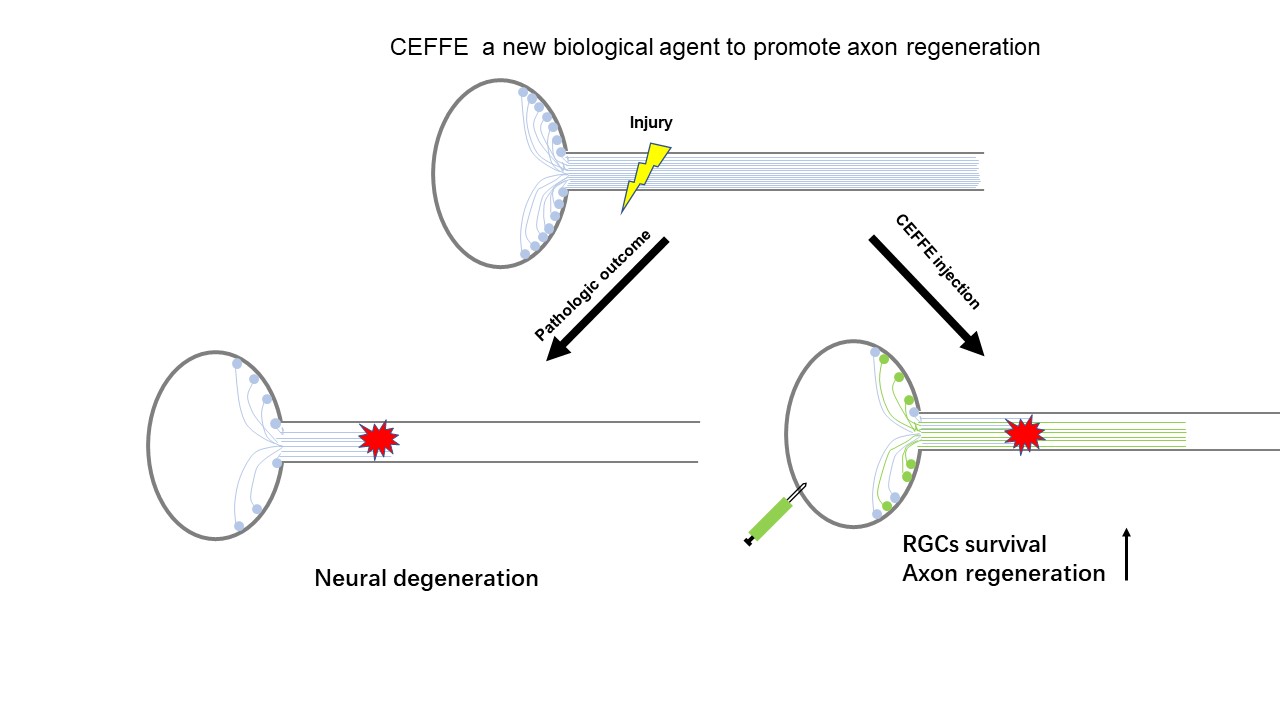

Supplement: Supplementary file 1 [file Image_1.JPEG]
