# Supplementary material for: Cell-free fat extract promotes axon regeneration and retinal ganglion cells survival in traumatic optic neuropathy
Source: Front Cell Neurosci. 2024 Mar 7;18:1344853. doi: 10.3389/fncel.2024.1344853 (PMC10954833; doi:10.3389/fncel.2024.1344853)
Supplement: Supplementary file 2 [file Table_1.DOCX]

Supplemental Table 1. Protein identified in CEFFE related to neurogenesis or axon genesis

| **Subcellular localization** | **Protein description** | **Gene name** |  | **Subcellular localization** | **Protein description** | **Gene name** |  |
| --- | --- | --- | --- | --- | --- | --- | --- |
| cyto_nucl | Copine-1 | CPNE1 |  | cytoplasm | Superoxide dismutase [Cu-Zn] | SOD1 |  |
|  | Dihydropyrimidinase-related protein 2 | DPYSL2 |  |  | Protein S100-B | S100B |  |
|  | Signal transducer and activator of transcription 3 | STAT3 |  |  | ADP-ribosylation factor 1 | ARF1 |  |
|  | Cyclin-dependent kinase 4 inhibitor C | CDKN2C |  |  | Tyrosine-protein phosphatase non-receptor type 11 | PTPN11 |  |
|  | COP9 signalosome complex subunit 2 | COPS2 |  |  | D-3-phosphoglycerate dehydrogenase | PHGDH |  |
|  | Platelet-activating factor acetylhydrolase IB subunit alpha | PAFAH1B1 |  |  | Serine/threonine-protein phosphatase 2B catalytic subunit alpha isoform | PPP3CA |  |
|  | Abl interactor 1 | ABI1 |  |  | Vinculin | VCL |  |
|  | Protein NDRG1 | NDRG1 |  |  | Myc box-dependent-interacting protein 1 | BIN1 |  |
|  | Acyl-protein thioesterase 2 | LYPLA2 |  |  | Nucleoside diphosphate kinase B | NME2 |  |
| extracellular | Laminin subunit beta-2 | LAMB2 |  |  | Peptidyl-prolyl cis-trans isomerase FKBP4 | FKBP4 |  |
|  | Epidermal growth factor receptor | EGFR |  |  | Ras-related protein Rab-10 | RAB10 |  |
|  | Mesencephalic astrocyte-derived neurotrophic factor | MANF |  |  | Aspartoacylase | ASPA |  |
|  | Clusterin | CLU |  |  | Growth factor receptor-bound protein 2 | GRB2 |  |
|  | Integrin beta-1 | ITGB1 |  |  | Mitogen-activated protein kinase 1 | MAPK1 |  |
|  | Galectin-1 | LGALS1 |  |  | Protein S100-A6 | S100A6 |  |
|  | Fibronectin | FN1 |  |  | EH domain-containing protein 1 | EHD1 |  |
|  | Palmitoyl-protein thioesterase 1 | PPT1 |  |  | Serine/threonine-protein kinase DCLK1 | DCLK1 |  |
|  | Laminin subunit beta-1 | LAMB1 |  |  | Ras-related C3 botulinum toxin substrate 1 | RAC1 |  |
|  | Beta-2-microglobulin | B2M |  |  | ADP-ribosylation factor 4 | ARF4 |  |
|  | Thy-1 membrane glycoprotein | THY1 |  |  | Protein arginine N-methyltransferase 5 | PRMT5 |  |
|  | Chondroitin sulfate proteoglycan 4 | CSPG4 |  |  | Pre-mRNA-processing factor 19 | PRPF19 |  |
|  | Apolipoprotein D | APOD |  |  | Septin-2 | SEPT2 |  |
|  | Alpha-N-acetylglucosaminidase | NAGLU |  |  | Rho GDP-dissociation inhibitor 1 | ARHGDIA |  |
|  | Mimecan | OGN |  |  | Dihydropyrimidinase-related protein 3 | DPYSL3 |  |
|  | 2',3'-cyclic-nucleotide 3'-phosphodiesterase | CNP |  |  | Myosin-10 | MYH10 |  |
| mitochondria | Dynamin-like 120 kDa protein, mitochondrial | OPA1 |  |  | Src substrate cortactin | CTTN |  |
|  | 2',3'-cyclic-nucleotide 3'-phosphodiesterase | CNP |  |  | Cell division control protein 42 homolog | CDC42 |  |
|  | Serine protease HTRA2, mitochondrial | HTRA2 |  |  | IST1 homolog | IST1 |  |
|  | Versican core protein | VCAN |  |  | Dynein light chain 2, cytoplasmic | DYNLL2 |  |
|  | Isocitrate dehydrogenase [NADP], mitochondrial | IDH2 |  |  | Myotrophin | MTPN |  |
|  | 2-oxoglutarate dehydrogenase, mitochondrial | OGDH |  |  | Kinesin-1 heavy chain | KIF5B |  |
|  | Cytochrome b-c1 complex subunit 8 | UQCRQ |  |  | Ubiquitin carboxyl-terminal hydrolase isozyme L1 | UCHL1 |  |
|  | NIF3-like protein 1 | NIF3L1 |  |  | Calcium/calmodulin-dependent protein kinase type 1 | CAMK1 |  |
|  | Trafficking protein particle complex subunit 4 | TRAPPC4 |  |  | Filamin-A | FLNA |  |
|  | Protein S100-A8 | S100A8 |  |  | Elongation factor 2 | EEF2 |  |
|  | Cofilin-1 | CFL1 |  |  | Spectrin beta chain, non-erythrocytic 1 | SPTBN1 |  |
|  | Ubiquitin-conjugating enzyme E2 variant 2 | UBE2V2 |  |  | 14-3-3 protein epsilon | YWHAE |  |
|  | Glutathione S-transferase P | GSTP1 |  |  | Cytoplasmic FMR1-interacting protein 1 | CYFIP1 |  |
|  | Apoptosis-inducing factor 1, mitochondrial | AIFM1 |  |  | Calcineurin subunit B type 1 | PPP3R1 |  |
| nucleus | Stathmin | STMN1 |  |  | Ras-related protein Rab-21 | RAB21 |  |
|  | Protein Hook homolog 3 | HOOK3 |  |  | Biogenesis of lysosome-related organelles complex 1 subunit 2 | BLOC1S2 |  |
|  | Serine/threonine-protein kinase PAK 2 | PAK2 |  |  | Transforming protein RhoA | RHOA |  |
|  | Caprin-1 | CAPRIN1 |  |  | Integrin-linked protein kinase | ILK |  |
|  | Drebrin | DBN1 |  |  | Band 4.1-like protein 3 | EPB41L3 |  |
|  | Ubiquitin-like modifier-activating enzyme 6 | UBA6 |  |  | 14-3-3 protein eta | YWHAH |  |
|  | Protein enabled homolog | ENAH |  |  | Twinfilin-2 | TWF2 |  |
|  | PDZ and LIM domain protein 7 | PDLIM7 |  |  | WD repeat-containing protein 1 | WDR1 |  |
|  | Echinoderm microtubule-associated protein-like 1 | EML1 |  |  | Protein S100-A9 | S100A9 |  |
|  | Vasodilator-stimulated phosphoprotein | VASP |  |  | Annexin A1 | ANXA1 |  |
|  | PDZ and LIM domain protein 5 | PDLIM5 |  |  | Hypoxanthine-guanine phosphoribosyltransferase | HPRT1 |  |
|  | Dystrophin | DMD |  |  | 14-3-3 protein gamma | YWHAG |  |
|  | Transcription factor p65 | RELA |  |  | Serine/threonine-protein phosphatase 2B catalytic subunit beta isoform | PPP3CB |  |
|  | EF-hand domain-containing protein D1 | EFHD1 |  |  | Alpha-soluble NSF attachment protein | NAPA |  |
|  | Spectrin alpha chain, non-erythrocytic 1 | SPTAN1 |  |  | Vesicle-associated membrane protein-associated protein A | VAPA |  |
|  | Phosphatidylinositol-binding clathrin assembly protein | PICALM |  |  | Ezrin | EZR |  |
|  | Microtubule-associated protein 4 | MAP4 |  |  | Ras GTPase-activating-like protein IQGAP1 | IQGAP1 |  |
|  | Microtubule-associated protein 1B | MAP1B |  |  | Actin-related protein 2 | ACTR2 |  |
|  | Focal adhesion kinase 1 | PTK2 |  |  | Ras-related protein Rap-1A | RAP1A |  |
|  | Golgin subfamily A member 4 | GOLGA4 |  |  | Mitogen-activated protein kinase 3 | MAPK3 |  |
|  | Na(+)/H(+) exchange regulatory cofactor NHE-RF1 | SLC9A3R1 |  |  | Serine/threonine-protein phosphatase PP1-gamma catalytic subunit | PPP1CC |  |
|  | Methyl-CpG-binding protein 2 | MECP2 |  |  | X-ray repair cross-complementing protein 5 | XRCC5 |  |
|  | Catenin alpha-1 | CTNNA1 |  | plasma membrane | Neurotrimin | NTM |  |
|  | Drebrin-like protein | DBNL |  |  | Dystroglycan | DAG1 |  |
|  | Serine/arginine-rich splicing factor 1 | SRSF1 |  |  | Plexin-B2 | PLXNB2 |  |
|  | Myotubularin-related protein 2 | MTMR2 |  |  | Integrin alpha-1 | ITGA1 |  |
|  | 5'-3' exoribonuclease 2 | XRN2 |  |  | Prolow-density lipoprotein receptor-related protein 1 | LRP1 |  |
|  | Transcriptional coactivator YAP1 | YAP1 |  |  | Receptor-type tyrosine-protein phosphatase mu | PTPRM |  |
|  | Reticulon-1 | RTN1 |  |  | Reticulon-4 | RTN4 |  |
|  | Microtubule-associated protein tau | MAPT |  |  | CD9 antigen | CD9 |  |
| cytoskeleton | Protein arginine N-methyltransferase 1 | PRMT1 |  | endoplasmic reticulum | Endoplasmic reticulum chaperone BiP | HSPA5 |  |
|  |  |  |  |  | Neuropilin-1 | NRP1 |  |
|  |  |  |  |  | Calreticulin | CALR |  |
|  |  |  |  |  | Lysosome membrane protein 2 | SCARB2 |  |
|  |  |  |  |  | C-Jun-amino-terminal kinase-interacting protein 4 | SPAG9 |  |
|  |  |  |  |  | Rab GDP dissociation inhibitor alpha | GDI1 |  |
|  |  |  |  |  | Beta-hexosaminidase subunit beta | HEXB |  |
