# Supplementary material for: Cell-free fat extract promotes axon regeneration and retinal ganglion cells survival in traumatic optic neuropathy
Source: Front Cell Neurosci. 2024 Mar 7;18:1344853. doi: 10.3389/fncel.2024.1344853 (PMC10954833; doi:10.3389/fncel.2024.1344853)
Supplement: Supplementary file 3 [file Table_2.DOCX]

Table 1 Gene primers used in the article.

| Mouse IL-1β-F | GAAATGCCACCTTTTGACAGTG |
| --- | --- |
| Mouse IL-1β-R | TGGATGCTCTCATCAGGACAG |
| Mouse IL-6-F | CTGCAAGAGACTTCCATCCAG |
| Mouse IL-6-R | AGTGGTATAGACAGGTCTGTTGG |
| Mouse iNOS-F | ACATCGACCCGTCCACAGTAT |
| Mouse iNOS-R | CAGAGGGGTAGGCTTGTCTC |
| Mouse TNF-α-F | CAGGCGGTGCCTATGTCTC |
| Mouse TNF-α-R | CGATCACCCCGAAGTTCAGTAG |
| Mouse IL-10-F | GCCCTTTGCTATGGTGTC |
| Mouse IL-10-R | TCTCCCTGGTTTCTCTTCC |
| Mouse ARG-F | CTCCAAGCCAAAGTCCTTAGAG |
| Mouse ARG-R | GGAGCTGTCATTAGGGACATCA |
| Mouse TGF-β-F | CCAGATCCTGTCCAAACTAAGG |
| Mouse TGF-β-R | CTCTTTAGCATAGTAGTCCGCT |
| Mouse CD206-F | TGGAGGCTGATTACGAGCAGT |
| Mouse CD206-R | TTGGTTCACCGTAAGCCCAAT |
| Mouse GAPDH-F | ATGGTGAAGGTCGGTGTGAA |
| Mouse GAPDH-R | TGAGTGGAGTCATACTGGAACA |
